# Big Earth data: disruptive changes in Earth observation data management and analysis?

**DOI:** 10.1080/17538947.2019.1585976

**Published:** 2019-03-14

**Authors:** Martin Sudmanns, Dirk Tiede, Stefan Lang, Helena Bergstedt, Georg Trost, Hannah Augustin, Andrea Baraldi, Thomas Blaschke

**Affiliations:** a Department of Geoinformatics – Z_GIS, University of Salzburg, Salzburg, Austria; b Italian Space Agency (ASI), Rome, Italy

**Keywords:** Digital earth, data access, satellite data portals, object-based image analysis (OBIA), remote sensing workflow

## Abstract

Turning Earth observation (EO) data consistently and systematically into valuable global information layers is an ongoing challenge for the EO community. Recently, the term ‘big Earth data’ emerged to describe massive EO datasets that confronts analysts and their traditional workflows with a range of challenges. We argue that the altered circumstances must be actively intercepted by an evolution of EO to revolutionise their application in various domains. The disruptive element is that analysts and end-users increasingly rely on Web-based workflows. In this contribution we study selected systems and portals, put them in the context of challenges and opportunities and highlight selected shortcomings and possible future developments that we consider relevant for the imminent uptake of big Earth data.

## 1. Introduction

It is widely agreed that Earth observation (EO) data and subsequently produced information are essential for understanding, modelling, and predicting natural processes, as well as the current and future state and dynamics of the human–Earth system. Regularly collected remote sensing and in-situ measurements are crucial sources for up-to-date knowledge about physical and human-related processes, if adequately exploited, and the two key technological elements of the Global EO System of Systems (GEOSS). However, directly generating knowledge about spatio-temporal patterns of human activities or natural processes is still a challenge, even with access to this increasing body of data. Directly observed can be specific physical aspects of entities as traces of evidence. This serves as proxies to monitor and assess goals and targets of major international frameworks, such as the United Nations Sustainable Development Goals (SDGs) (United Nations 2015a), the Sendai Framework for Disaster Risk Reduction (United Nations 2015b), the Paris Agreement on Climate Change (United Nations 2015c), or the New Urban agenda (Corbane et al. 2017; United Nations 2017). Indirect cues derived from remotely sensed data can provide evidence that serves a multitude of domains, including the domains of public health, human settlement observation and the entire human-environment nexus as addressed by the SDGs and Group on Earth Observations (GEO)/Committee on Earth Observation Satellites (CEOS) ‘Earth Observations in Service of the 2030 Agenda’.

While the utility of EO data is generally recognised, often such data cannot be directly translated into information because the data lack inherent semantic meaning: Generally, EO data require interpretation to some degree. This complicates recent developments in big data and the data-driven, machine-based information production, which is purely based on statistics. Not only is the variety of available data sources expanding, but so are applications accessing multiple data sources. The data volumes considered in some analyses are becoming too big to make a copy over the Web at the user’s computer and challenges the capacity of traditional workflows, often characterised by a high degree of human interaction and download-driven approaches. Broadly speaking, this may be characterised by the meanwhile well-known slogan, ‘bring the user to the data instead of the data to the user’. Data can be ‘big’ in different ways (Lynch 2008) where they can be associated with five different ‘V’s’, i.e. characterised by an extreme data volume, exhibiting a wide variety of forms, requiring a high velocity of data processing, and a need to deal with the veracity of data uncertainty (and, finally, turn the data into value).

When trying to particularise big Earth data within big data, we inevitably connect to the vision of ‘The Digital Earth’, coined in 1998 by then US vice president Al Gore. Since then, many elements of this vision have been implemented and become a reality, such as GEOSS; others have emerged in an unprecedented and rather unpredictable way (e.g. social media networks). Guo et al. (2017) identified the emergence of a new stage when the vision of the Digital Earth entered into the era of big data, turning the original concept into what they call big Earth data. While there is no community-agreed definition of (scientific) big Earth data, some particular characteristics include:

- Non-repeatability: observations of physical objects and processes are unique in space and time and generally cannot be repeated
- Uncertainty: big data involves different approaches to observation and recording, as well as indirect observation and sampling
- Multi-dimensionality: a wide range of data sources and complex analysis methods including cross-scale modelling lead to a wide range of dimensionality and often multi-dimensionality
- Computational complexity: combining the first three issues results in a high degree of computational complexity in data analysis.

In recent years, technological developments from other areas have been transferred to the remote sensing world, producing a somewhat revolutionary effect across the spectrum of activities in routine applications and science. Data cube concepts and data cube technologies have recently gained popularity, but were developed in domains outside EO data (Nativi, Mazzetti, and Craglia 2017). Next to online download portals, such as the USGS Earth Explorer and the Copernicus Sentinel Hub, analytically oriented solutions such as Google Earth Engine (GEE) (Gorelick et al. 2017) or EarthServer (Baumann et al. 2016) are now used by professional communities. This change is directly linked to the provision of free or at least inexpensive data associated with the launch of new EO satellites and access to government data.

**Figure 1.**
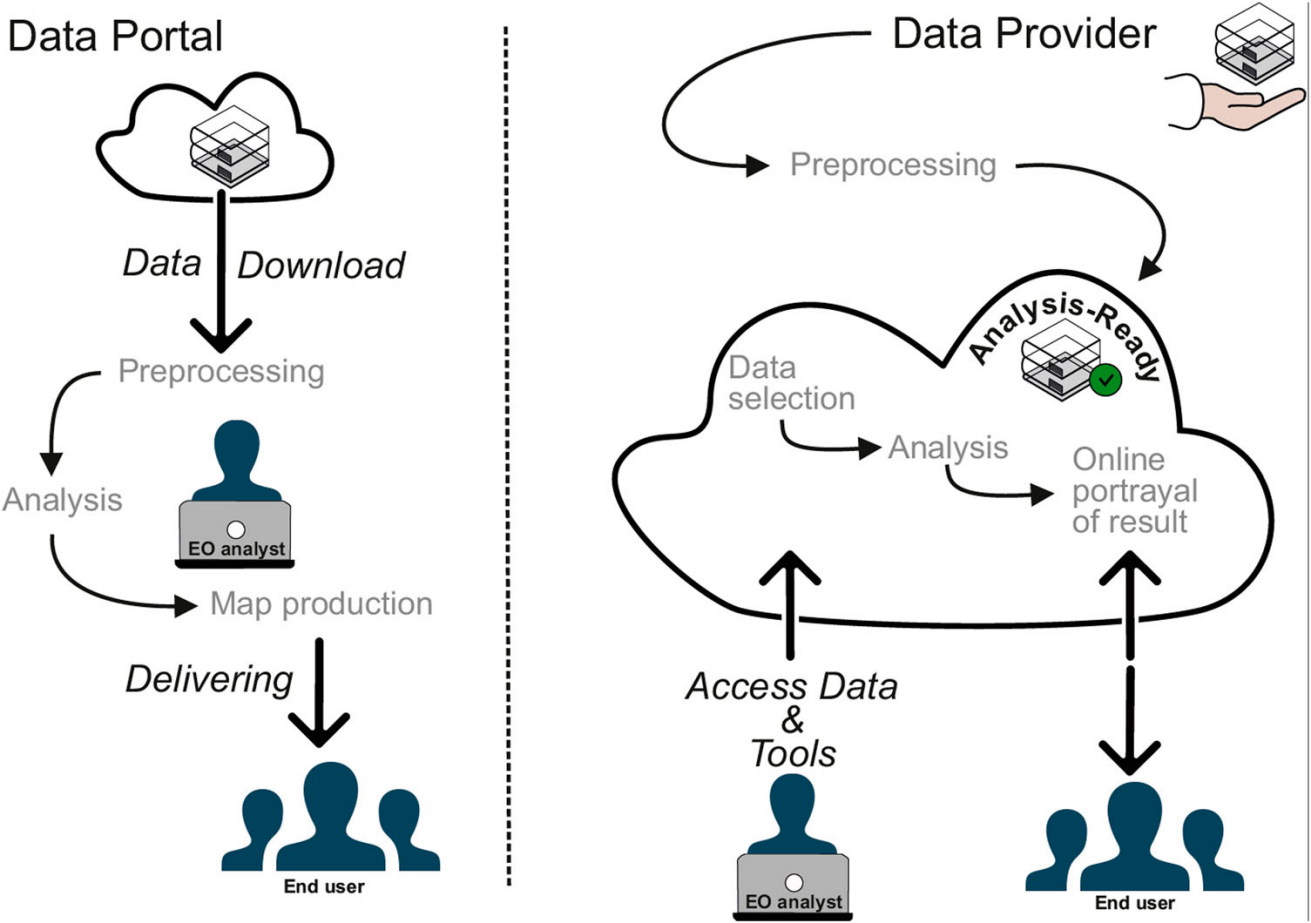
Changes in EO data analysis workflow. The left part shows the ‘traditional’ way of information production where the complete dataset is downloaded, analysed locally and transformed into a map, which is delivered to the end user in a one-way road. The right side shows an envisioned and partly already implemented workflow, where the data provider generates analysis-ready data in a cloud environment where EO analysts can access them together with their own or already existing tools. The end product will also reside within the cloud environment, which can be accessed by the end user. The interactive elements might be a simple application of geographical or temporal filters and colour selection, or even the combination with other datasets.

Although these parts of the EO data analysis workflow are relatively new or utilise newer Web-based opportunities when compared to the 1990s, the dominating strategy is still locally processing downloaded data sets. The enormous increase of data, proliferation of cloud service architectures and opportunities of state-of-the-art Web technologies, now make it possible for users to more easily access remote sensing data. This trend is likely to continue, but the data volumes used in the analysis will be a limiting factor as long as the processing in EO data workflows continues to occur locally in the client. Thus, there is a need for huge technological progress in big Earth data analysis, or even a complete, disruptive change in workflows as illustrated in Figure 1.

**Figure 2.**
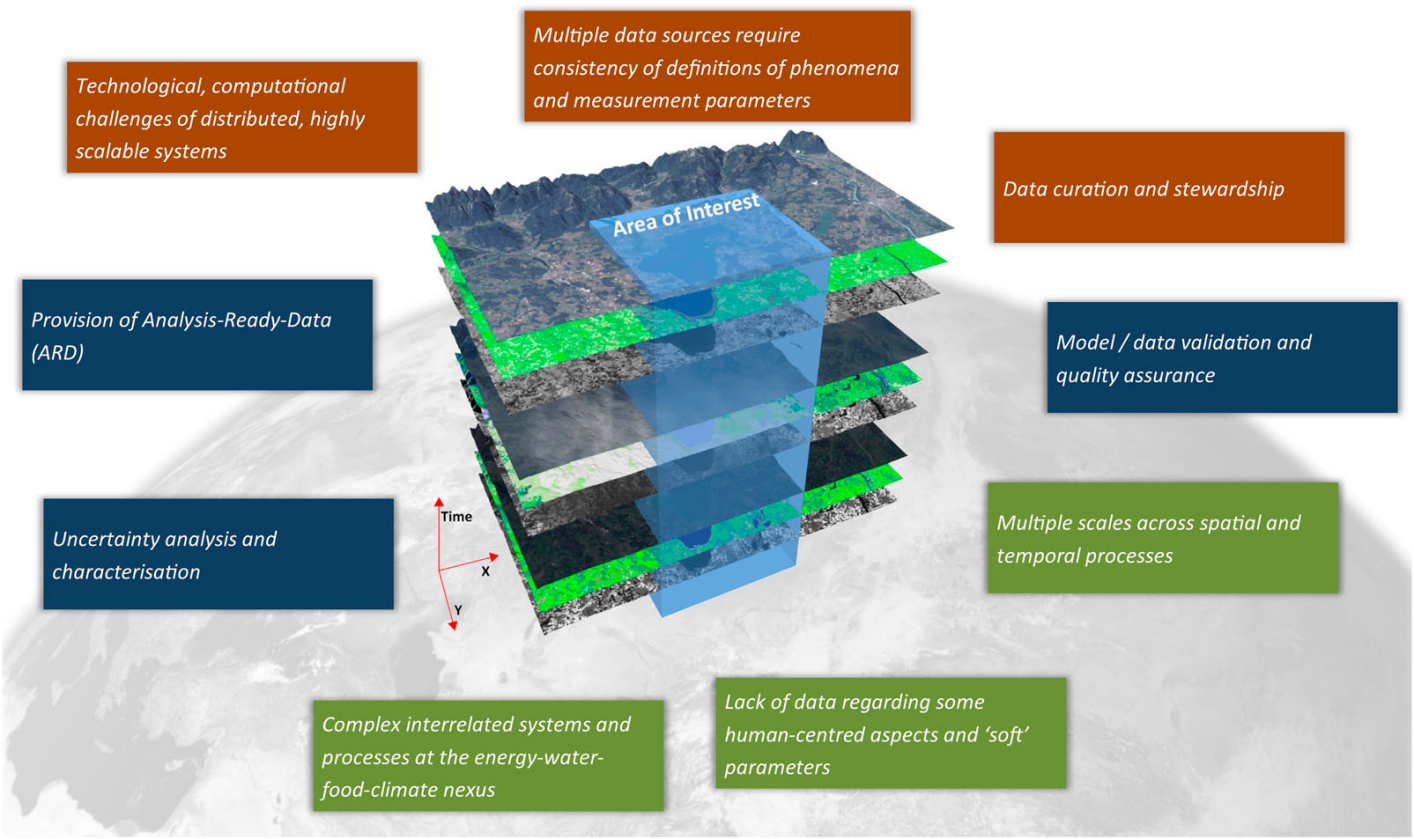
Some key challenges attached to big Earth data encompassing technological (orange), methodological (blue) and societal (green) aspects. Own composition based on Tiede et al. (2017).

The opportunities of big Earth data pose several challenges. The most obvious is how to handle the increase in data volume. This may be illustrated by the opening of the USGS Landsat archive and the free distribution strategy (Wulder et al. 2012), along with the advent of the openly accessible Sentinel satellite data as part of the Copernicus programme of the European Commission and ESA (European Commission 2013). For example, the optical Sentinel-2 A and B satellites alone produce already ∼3.4 TB of data on average per day according to the acquisition plan (Copernicus Space Component Mission Management Team 2018), the combined fleet of Sentinel-1, Sentinel-2 and Sentinel-3 produce an estimated data volume of ∼20 TB per day (Esch et al. 2018). However, next to the massive amount of free data many more challenges exist and are exemplarily illustrated in Figure 2.

In this paper we argue that the unprecedented data volume, along with its variety, velocity and other V’s necessitate more than just a quantitative adaptation, but rather disruptive changes and a shift in the way how users interact with data and produce information. The remainder of the paper is structured as follows: Section 2 gives an overview of existing systems and solutions; Section 3 puts them in context of big Earth data challenges; in Section 4, selected future developments are highlighted and explained; and the paper closes with some concluding remarks in Section 5.

## 2. Overview of existing systems for big Earth data management and remote processing

Evolutions in spatio-temporal resolutions of remote sensing technology, an increasing number and variety of EO satellites, the adoption of open data policies and increased demand on the application side have led to innovative approaches in storing, managing, processing, and analysing EO data commonly used today. The base-technologies include distributed multidimensional array databases such as SciDB (The SciDB Development 2010) or Rasdaman (Baumann et al. 1997), and tools and libraries with features for array processing such as the GEE (Gorelick et al. 2017) or the Open Data Cube (ODC) (Open Data Cube Initiative 2017). Many of the new systems and portals based on these technologies have the common aim to enable users to perform their analysis tasks in a Web-based environment (Esch et al. 2018). The systems and portals listed in this chapter focus on Web-based data access or processing, and are currently operational and in use. They are categorised as public, private, or public–private partnerships (PPP), and by their degree of interactivity. Relevant norms, standards and guidelines are mentioned prior to the technical systems.

### 2.1. Norms, standards and guidelines in the context of EO data storage and processing

To deal with new, emerging and ever-changing systems regarding EO data, several norms, standards and guidelines have been set and agreed upon by the community. The Quality Assurance Framework for Earth Observation (QA4EO, endorsed by CEOS as a contribution to facilitate the GEO vision for a GEOSS) strives to promote synergistic use of data derived from a multitude of EO systems (satellite, airborne and in-situ measurements) as well as assure high quality of data and data products (Fox 2010). Within the GEO Strategic Plan 2016–2025 (GEO 2015) GEOSS Data Management Principles were formulated outlining five main key words: discoverability, accessibility, usability, preservation, and curation. The Research Data Alliance (RDA) provide ongoing collection and analyses on state-of-the-art data cubes and array databases in particular, including technical reports, standards, and implementations.

Standards encompassing data storage, data processing, network requirements and Web applications are set up by the Open Geospatial Consortium (OGC) (Open Geospatial Consortium). Some of them especially address online processing of EO data (Petcu et al. 2010; Wagemann et al. 2018), namely the Web Coverage Processing Service (WCPS) (OGC 2009) and the Web Processing Service (WPS) (OGC 2018). They bring new opportunities to access big EO data via the Internet and to process them on the server-side. Further efforts are on the way, for example, the recently executed OGC Testbed 13 with a (sub-)focus on ‘Cloud Computing Environment for Earth Observation Data’ (Percivall 2017), or to standardise data cubes as OGC coverage data type (Baumann 2017a). The OGC Big Data Domain Working Group aims to provide best practices for big data interoperability, access, and analytics (OGC Big Data DWG 2018). To the best of the authors’ knowledge, specific standards for big Earth data Web-based processing have not yet been developed (Big Data Value Association 2017), including missing standards for the application of algorithms across platforms, multi-level processing, uncertainty in pre-processing, times series analysis and, especially, a commonly agreed definition of analysis ready data (ARD) (Egorov et al. 2018; USGS 2018).

In a more practical sense, to be able to allow ‘time first, space later’ as well as ‘space first, time later’ approaches, pre-processing of (optical) data to ARD, e.g. ESA Level 2A, is necessary. ESA EO Level 2 data are surface reflectance values of optical images, which have been corrected for atmospheric, topographic and adjacency effects and are accompanied with a scene classification map (SCM). Generating an SCM requires an EO image of high radiometric quality, while SCM is mandatory to better condition common pre-processing problems. Similar to vision itself, generating ESA EO Level 2 data is an ill-posed problem in the Hadamard sense, where only a few solutions exist or were proposed (Baraldi et al. 2017; Sen2Cor 2017). In the case of Sentinel-2, only small parts of the archive are being systematically processed up to this level, but it is the responsibility of the EO data provider to deliver a high-quality EO Level 2 data at the ground segment as a building block for further activities and the production of ARD. Once a high-quality building block is finally made available for free in the upstream, then in the downstream users are much better posed for complex analyses.

Instead of only relying on mapping accuracy, solutions can be tested against a well-defined set of outcome and process quality indicators, e.g.:

- accuracy (e.g. mapping accuracy);
- efficiency in computation time and memory occupation;
- costs of labour and computer power;
- degree of automation as contrast to degree of user interaction;
- timeliness, defined as the time interval between data acquisition and product generation;
- robustness to changes in input data;
- robustness to changes in input parameters, if any;
- scalability to changes in sensor and user specifications.

Having a set of quality indicators such as these would guide algorithm and workflow design for high-quality products and services required by end-users of EO-derived information. These indicators do not only stress the quality of the products (e.g. thematic accuracy), but also the quality of the workflow that is used to generate the product.

### 2.2. State-of-the-art technical solutions

**Figure 3.**
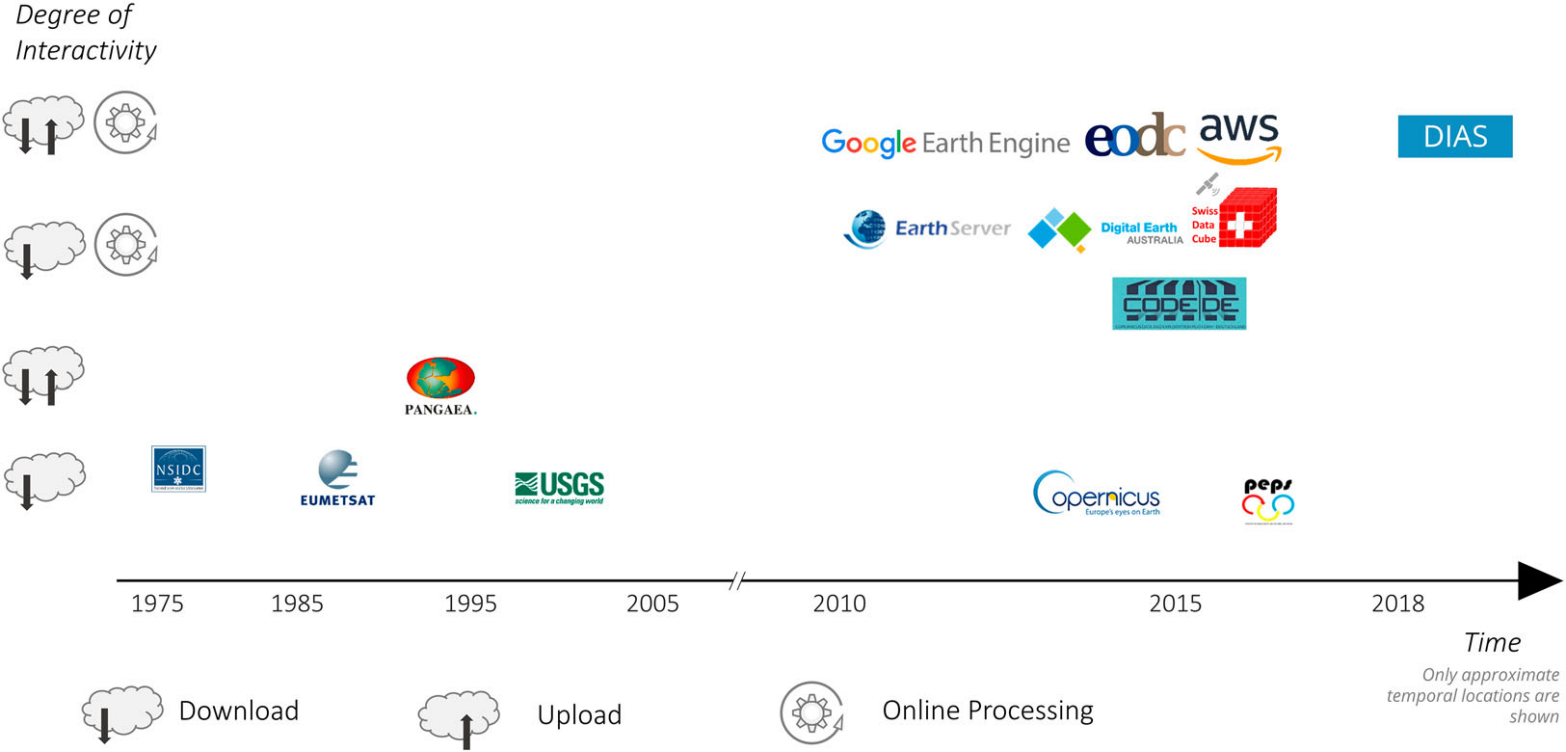
Timeline of technical solutions and their degree of interactivity (e.g. online processing, up- and downloading of data). For a detailed description see Table 1.

Different stakeholders have developed many practical systems and portals allowing storage, processing and analysis of big Earth data and made them available. While detailed technical discussions can be found in recent literature, e.g. in Baumann et al. (2018a), we focus on overall trends in the historic context. Figure 3 illustrates the timeline of the emergence of different EO portals (see Table 1 for details), some of which utilise the aforementioned technologies, and clearly shows the increase in the number of available portals and solutions over time. The number of platforms with higher interactivity, meaning the number of possibilities for the user to interact with the service (e.g. online processing, up- and download of data), has clearly increased in recent years. The degree of interactivity strongly shapes the users experience of a platform because it dictates the degree to which a service can be integrated into the workflow and is, therefore, an important characteristic.

**Table 1.** Overview of available systems and solutions dealing with Big Earth data.

Name	Funding	Data structure	Available data	Geographic coverage
Google Earth Engine	Private	Container of 2D gridded raster bands	Satellite Imagery; Satellite-derived data products	Global
Amazon Web Services	Private	Image files	Satellite Imagery; Satellite-derived data products	Global
Earth Server	Private/public	Data cube	Satellite Imagery; Satellite-derived data products; Model outputs	Global
EODC	Private/public	Image files	Satellite Imagery; Satellite-derived data products	Global
Swiss Data Cube	Public	Data cube	Satellite Imagery; Satellite-derived data products	Switzerland
Digital Earth Australia	Public	Data cube	Satellite Imagery	Australia
CODE-DE	Public	Image files	Satellite Imagery	Global
PEPS	Public	Image files	Satellite Imagery	Global
Earth Explorer	Public	Image files	Satellite Imagery; UAS Imagery; Data Products; Digital Maps	Global
Copernicus Open Access Hub	Public	Image files	Satellite Imagery	Global
EUMETSAT Data Centre	Public	Image files	Satellite Imagery; Satellite-derived data products	Global
NSIDC	Public	Raster data; point data; vector data	*In situ* data sets; Model outputs; satellite-derived data products	Global (specific focus on polar regions)
PANGEA	Public	Raster data; point data; vector data	Satellite-derived Data Products; Model outputs	Global

#### 2.2.1. Private company initiatives

A popular solution provided by a private company is GEE (Gorelick et al. 2017; Patel et al. 2015). In GEE the most common remote sensing data (e.g. Landsat, Sentinel and MODIS) are available and can be processed without requiring download. Users can also upload their own private data. A Web-based integrated programming environment (IDE), as well as an application programming interface (API), can be used to perform pixel-based image processing. GEE has been used in global studies, such as in Pekel et al. (2016) or in Hansen et al. (2013).

Another private solution is provided by the Amazon Web Services (AWS, https://aws.amazon.com/earth). AWS offers users access to a variety of datasets (e.g. Landsat, Copernicus, MODIS, digital elevation models (DEM)) in a cloud-based environment (Palankar et al. 2008). Services like the EO Browser (https://apps.sentinel-hub.com/eo-browser/) provided by the company Sinergise are based on AWS.

#### 2.2.2. Public–private partnerships

The EarthServer and EarthServer-2 is a FP7/H2020 funded research project applying the array database Rasdaman as storage and processing engine (Baumann et al. 2016). EarthServer-1/2 transforms the data into a data cube by facilitating Rasdaman capabilities for access and processing while adopting existing standards (Baumann et al. 2016). Data can be filtered, queried, extracted and processed using OGC WPS and WCPS.

The Earth Observation Data Centre for Water Resources Monitoring (EODC) specifically targets Earth observation and geographic information applications and users. It was one of the first data centres which provides computational resources and software directly where its data is stored (Wagner et al. 2014). The EODC offers access to a variety of data, most notably Copernicus data, in a cloud environment with special focus on tailored solutions for non-specialist users, like non-governmental organisations (NGOs) or governmental agencies (Wagner et al. 2014). The centre’s capabilities have been used in a variety of studies, such as using Sentinel-1 Synthetic Aperture Radar (SAR) data to retrieve geophysical parameters (Naeimi et al. 2016).

The Copernicus Data and Exploitation platform – Germany (CODE-DE, https://code-de.org) is a national access tool, geared towards national and local authorities, research institutions as well as companies and private persons, specifically in Germany. The CODE-DE platform offers users access to the data of the operational Sentinel satellites as well as the Copernicus services. Being solely a storage system at the moment, capabilities for processing and analysing data are planned (https://code-de.org/en/about as of 2 July 2018).

The Plateforme d’Exploitation des Produits Sentinel (PEPS) platform (https://peps.cnes.fr/rocket) is the French mirror site for the distribution of Sentinel products. It was launched in 2015 by the CNES (https://peps-mission.cnes.fr/en/peps-0 as of 2 July 2018). This platform provides free access to data sets collected by the Sentinel satellites and allows users to search, select and download data. PEPS is the French counterpart to the CODE-DE platform, and is designed to encourage wide use of available satellite and Copernicus products.

#### 2.2.3. Public initiatives

Many space agencies and research facilities provide their collected data sets directly to end-users, providing only search and download functionality. Examples include the Copernicus Open Access Hub of the ESA (https://scihub.copernicus.eu) and the EUMETSAT Data Centre of the European Organisation for the Exploitation of Meteorological (https://eoportal.eumetsat.int). The EarthExplorer, GloVis, and the USGS’s LandsatLook Viewer all provide access and/or visualisation of Landsat products (U.S. Geological Survey). The NASA sensor Web suite consists of software tools for accessing, processing and analysing data. The system allows the combination of satellite, *in situ*, and UAV data sets (Delin et al. 2005). In 2017, the European Commission started developing the Copernicus Data and Information Access Services (DIAS), publicly launched in June 2018. These systems not only allows access to data sets but also provides processing resources and tools for data analytics and will – once established – most likely turn into a PPP organisational structure.

Numerous smaller solutions geared towards local or regional applications exist in different research domains. Data sets covering the cold regions of the Earth are provided by the National Snow and Ice Data Center (NSIDC) (Barrett 2003; Scharfen et al. 2000). Available products range from *in situ* data sets (in form of point measurements) to raster data sets based on satellite data (e.g. soil moisture based on measurements from the Soil Moisture Active Passive (SMAP) satellite (Entekhabi et al. 2010), near-real-time sea ice coverage based on the Special Sensor Microwave Imager/Sounder (SSMIS) radiometer system (Sun and Weng 2008). Coverage ranges from local to global. Another example from the field of earth and environmental science is the PANGEA information system, which functions as an Open Access library for a large variability of data products (including data sets based on satellite imagery).

## 3. Gaps and challenges: technology, policy, science and society

In the last years, it has become increasingly apparent that the state-of-the-art technology for big Earth data emphasises Web-based processing instead of download-driven approaches. This shift comes with several challenges, but also opportunities for the entire remote sensing community in general and single users in particular. Earlier, scientific methods were restricted to the approach of starting with a hypothesis and testing it for consistency by specifically collecting a statistically sufficient amount of data. In the big Earth data era, with its continuous and application-independent data acquisition, new data-driven approaches are emerging where statistical relations are more important than causality. Some even claim that there is an ‘end of science’ or ‘end of theory’ with an ongoing discussion about it (Mazzocchi 2015). In this context, Boulton (2018) argues that effects of big Earth data can be compared to the invention of the microscope, allowing to see complex but previously concealed patterns. Others argue that biased data require biased methods and advocate for including semantics in data analysis, especially when data from multiple sources are combined (Narock and Shepherd 2017; Scheider, Ostermann, and Adams 2017). Scientific discussion aside, in practical applications, user communities of big Earth data will be further broadened to non-expert user groups seeking practical answers in the data. Having investigated the state-of-the-art in EO data, the ‘triple’ of (1) accessible and analysis-ready data, (2) Web-based tools and (3) sharing results was selected in this contribution to discuss the current dynamics in the field of big Earth data.

### 3.1. Data access

The open access policy of many scientific EO data, e.g. from Landsat and Copernicus allow analyses at a continental or even global scale. Simultaneous to the political decisions, requirements are increasing both on the user side to execute ‘any query, anytime, on any size’ (Baumann 2017b, 2018) and on the data provider side ‘to bring the user to the data, not the data to the user’. Public solutions, as well as private ones, react with a variety of new and innovative tools, which have been recently developed (e.g. DIAS, ODC, EarthServer, EO Browser, GEE).

The traditional approach of downloading image data to personal storage devices and performing the tasks on individual infrastructure and software is time-consuming and inefficient when dealing with large amounts of remote sensing data. In the EO domain, data become increasingly ‘immobile’ (i.e. data downloads are cumbersome or not feasible any more), requiring adequate tools for online access and processing within the environment where the data is located. Due to technological advancements in digital infrastructure, increased computing power and storage capabilities, many computational intensive studies are using cloud computing, where data and software can be accessed on demand, without the need of owning and maintaining physical hardware.

New means of accessing data in the context of big Earth data is driven by the aim to provide ARD in a Web-based accessible high-performance computing (HPC) facility. To date, there is no clear common understanding in the community of ARD or the optimal software technology to facilitate access to data and tools. Individual or institutional interpretations of ARD currently comprise concepts ranging from recommendations of certain pre-processing steps to convert data from original digital numbers into a physical units (e.g. surface reflectance) up to the generation of ESA EO Level 2 products, following the definition by ESA (Baraldi et al. 2017). For example, the CARD4L CEOS definition (Killough 2016) refers to a recommendation of pre-processing to surface reflectance. The EO Level 2 definition promoted by ESA refers to image data provided in physical units of measurement, corrected for topographic, atmospheric and adjacency effects together with an SCM. The SCM is a pre-requisite for proper image correction but often not delivered in conjunction with the corrected data sets, such as for Landsat Level 2 products (Baraldi et al. 2017). Furthermore, the SCM can be used to query image content directly or can serve as starting point for further analysis (Tiede et al. 2017). Currently, ESA Level 2 products are generated from Sentinel-2 imagery at the ground segment only for selected areas, e.g. the main land of Europe, a worldwide provision is planned (ESA 2018).

Besides technical challenges, organisational and political challenges exist and are partly unsolved or not even discussed. For a research organisation or a company without on premise hardware infrastructure, questions arise whether the data and the developed algorithms are stored securely enough. In the case of open science, where everything is aimed to be open access, this might not be relevant, but for business models, this can be a serious risk. On a political level, questions might arise whether the data should be stored in a central place or replicated at the country-level. The storage of data at a single, central entity, potentially located in another country, and thus potentially outside their influence, might not be politically desired by some countries, institutions or organisations. Data providers, on the other hand, are increasingly being forced to transform themselves into cloud providers (e.g. Copernicus with DIAS, DigitalGlobe with GDB-X) and face new challenges on their own. Examples include how to finance increased costs, challenges regarding IT security when users are uploading and executing their own code (e.g. within Docker containers), but also questions regarding neutrality – or not – and non-discrimination of different user types.

### 3.2. Processing and analysing

In recent years, the term EO data cube (or geospatial data cube, sometimes data cube only) has emerged to describe a new solution to store, organise, manage and analyse EO data (Baumann et al. 2016, 2018b; Giuliani et al. 2017; Purss et al. 2015). EO data cube technology is tightly linked to the aforementioned term ARD (Baumann 2017a, 2017b; Giuliani et al. 2017; Lewis et al. 2016, 2017). Different data cubes have been developed and different initiatives cover various scales, from regional to global. They provide different data sets, from single data sources to multiple data sources differing in spatial and temporal resolution, and use different infrastructures, software implementations and user interfaces (Baumann et al. 2018b). Examples of EO data cubes include the aforementioned EarthServer, the Swiss Data Cube (SDC) (Giuliani et al. 2017), and Digital Earth Australia, formerly known as the Australian Geoscience Data Cube, AGDC) (Dhu et al. 2017; Lewis et al. 2016, 2017). In contrast to traditional storage modes of EO data using the images-as-a-temporal-snapshot storage model, in a data cube, the data can be ordered along at least one non-spatial axis (e.g. time) in addition to the spatial axes. Therefore, data cubes store data access-oriented rather than acquisition-oriented and facilitate different access types within one data structure (e.g. spatial, temporal, and thematic).

Data cubes represent one of the emerging and efficient techniques for storing and analysing big Earth data. These multi-dimensional arrays can speed up the performance of trimming, slicing or extracting information from gridded data stored in many dimensions, including spatial or temporal (Baumann 2017b). Compared with classic storage of gridded data optimised for horizontal (‘spatial’) access, the data cubes are opening a new path towards better performance in time series analysis, when the vertical (‘temporal’) access to the gridded data is needed (Baumann 2017b). Time series analysis is able to show patterns of phenomena through time and help improve understanding of the environment.

Making big Earth data processing faster relies on reducing the amount of input data or using parallel computing, which has become routine in big Earth data analytics. The process of dividing larger tasks into smaller ones that are distributed and performed simultaneously on multiple computing sources is difficult if data dependencies within the task exist. However, if applicable, it results in a decrease of financial expenses and better usage of existing infrastructure, as reported by Kempeneers and Soille (2017). During the design and application of algorithms, one has to keep in mind that many algorithms work well in ‘small data’ analytics, but few work well in ‘big data’ analytics. Only a small subset is sufficiently suited for both conditions. Next to the accuracy and correctness of the result, the workflow itself needs to be accompanied by the aforementioned quality indicators (Baraldi et al. 2017). The community has not yet developed or agreed upon a full and descriptive set of quality indicators for both products and workflows.

The backbone of big Earth data analytics is some type of distributed computing platform (e.g. clouds). Cloud computing services are broadly categorised into four types: Infrastructure as a Service (IaaS), Platform as a Service (PaaS), Software as a Service (SaaS) and Data as a Service (DaaS) (Yang et al. 2011). IaaS provides virtualised computing resources over the internet that can be used for storage, backup or big data analytics, with a pay-as-you-go structure. PaaS provides the hardware and software tools over the internet needed for applications development, testing and delivering. Microsoft Azure and Google App Engine are some of the most popular PaaS. SaaS is often referred to as ‘on-demand-software’, where cloud providers deliver software applications over the Internet, usually on a subscription basis. Common examples of SaaS include the ArcGIS Online (ESRI) cloud implementation, Microsoft Office or Google Gmail and apps. DaaS provides access to data discovery, access, and utilisation, including the software needed to interpret the data (Olson 2009; Yang et al. 2011).

While Web-based online processing (e.g. in a cloud environment) is becoming a well-adopted solution for processing big Earth data, it also comes with new challenges that need to be addressed accordingly. For example, if data storage is distributed on several clouds, one still has to move the data to use or share them if no standardised interface is provided. Additionally, the service providers’ infrastructure raise concerns regarding disruptions, such as due to a power outage (Marx 2013), as well as security and privacy concerns. A well-known issue in geospatial analysis is represented by interoperability and portability of data and methods (e.g. complex, multiple data formats, or lack of standardisation). This is also being propagated in Web-based services, where users might be ‘locked in’ (i.e. tied to a specific cloud provider).

All of the challenges represent a barrier in a wider adoption of this technology and ways of dealing with these challenges need to receive important consideration in the future. Web-based platforms are still not easily accessible for researchers. Main obstacles include low programming skills, constraints regarding security, or vendor lock-in. Cloud computing is the facilitator of big Earth data analyses with larger spatial and temporal extents and the ever-growing data volumes. However, working towards wider openness and user friendliness is an ongoing challenge.

### 3.3. Information sharing

The interdisciplinary opportunities of big Earth data have grown nearly as fast as the data amount. So far, big Earth data were mainly used as a standalone product for the observation of physical Earth dynamics, such as climate change (Ford et al. 2016; Karpatne and Kumar 2017) or land-use and land-cover (Lewis et al. 2016; Mack et al. 2017). Recently, social media data were added and combined with satellite-derived products not only for social studies (Corbane et al. 2017), but also to fill gaps in remotely sensed data availability (Cervone et al. 2017; Panteras and Cervone 2016). A study itself has no value if the information is not generated and shared at the right time with the right people. Besides scientific papers, other forms of sharing results are becoming increasingly important, including accessibility and comprehensive portrayal, such as in a Web-based environment.

The sheer amount of multi-dimensional and multi-temporal data, interdisciplinary research, and the dynamics of new developments, in general, makes sharing results, algorithms and knowledge between the experts of different fields and non-expert users essential. This becomes more relevant when the integration of non-EO data (e.g. socioeconomic data) and EO data significantly increases the complexity of interpretation (Guo et al. 2014; Guo et al. 2017). One of the most pressing questions arises from the availability and accessibility of processed data (i.e. under which conditions can data be combined and uploaded to external storages? When and how should the results be shared and published?).

Although the cloud facilitates integration of multiple datasets (e.g. commercial or open data) their combined processing might be subject of legal consideration. The ownership of the source data, as well as the processed products derived from data, must be completely clear. Even if licenses for open data explicitly define the framework for data usage, this might not necessarily be the case for products derived from these data, which is not always documented and easily accessible. Furthermore, although the obstacles for sharing results are lowered, especially for new users without expert knowledge, and visibility and usability of results is increased, the scientific peer-review publication system can prevent a comprehensive ‘open results’ approach for unpublished data. At the technical side, risks of vendor lock-in and non-transferability of data and results exist. In particular, cloud providers might decide to turn off their service, leaving users and their data in an unclear and problematic state. An example from outside the EO domain is the Google Code Project Hosting service (DiBona 2015).

The societal impact of using big Earth data is growing rapidly and contributing to a more transparent society for consumers. Where big data meets the society the privacy concerns for consumers are evoked, if technological developments are not properly accompanied by clear legislation. Employing big data technologies for societal benefits will lead to better strategic and operational decisions, like efficient environmental monitoring, assessment of climate change impacts or efficient territorial planning of urban and rural settlements. A lot of potential can be found if big Earth data analyses are tied to SDGs and other global initiatives. It may also foster and create a diverse commercial EO market for products and services.

To overcome the challenges mentioned in this chapter, a clear ethical guideline for the acknowledgement of previous work and sharing of results is needed. For instance, a clearly defined and less restrictive open data policy might not only improve the scientific workflow, but also the reproducibility and transferability of results. The positive effects of unrestricted access to ARD might yield another increase of EO data uptake, similar to the significant increase of Landsat image use after the opening of the archive. However, reproducibility not only requires a certain type of data but also semantic annotation of workflows (Scheider, Ostermann, and Adams 2017). This ensures data stewardship, comprehensive data lineage and trust in results. Producers of products and services are asked to disseminate detailed documentation of the data and workflows together with their results.

The essential requirement to assist the user in information sharing is especially an easy accessibility and efficient query system for raw data, data products and their lineage. Especially users without a profound knowledge of EO, such as in interdisciplinary research, should also be able to benefit from the availability of already processed products. Similarly, the provider of the cloud environment is very rarely the actual data collector. The acquisition of data and different processing steps should, therefore, be well documented and comprehensible for the user. In addition, sharing and communicating errors, uncertainties and pitfalls must occur to avoid inappropriate data use that ends up in misconception.

## 4. Possible future directions

While it can be concluded that big Earth data as a topic has arrived in the remote sensing community and that specific future developments seem to be assured, there is an ongoing discussion about their directions. This paper only considered imaging sensors, and while not a synonym of big Earth data, they make up a large portion.

**Figure 4.**
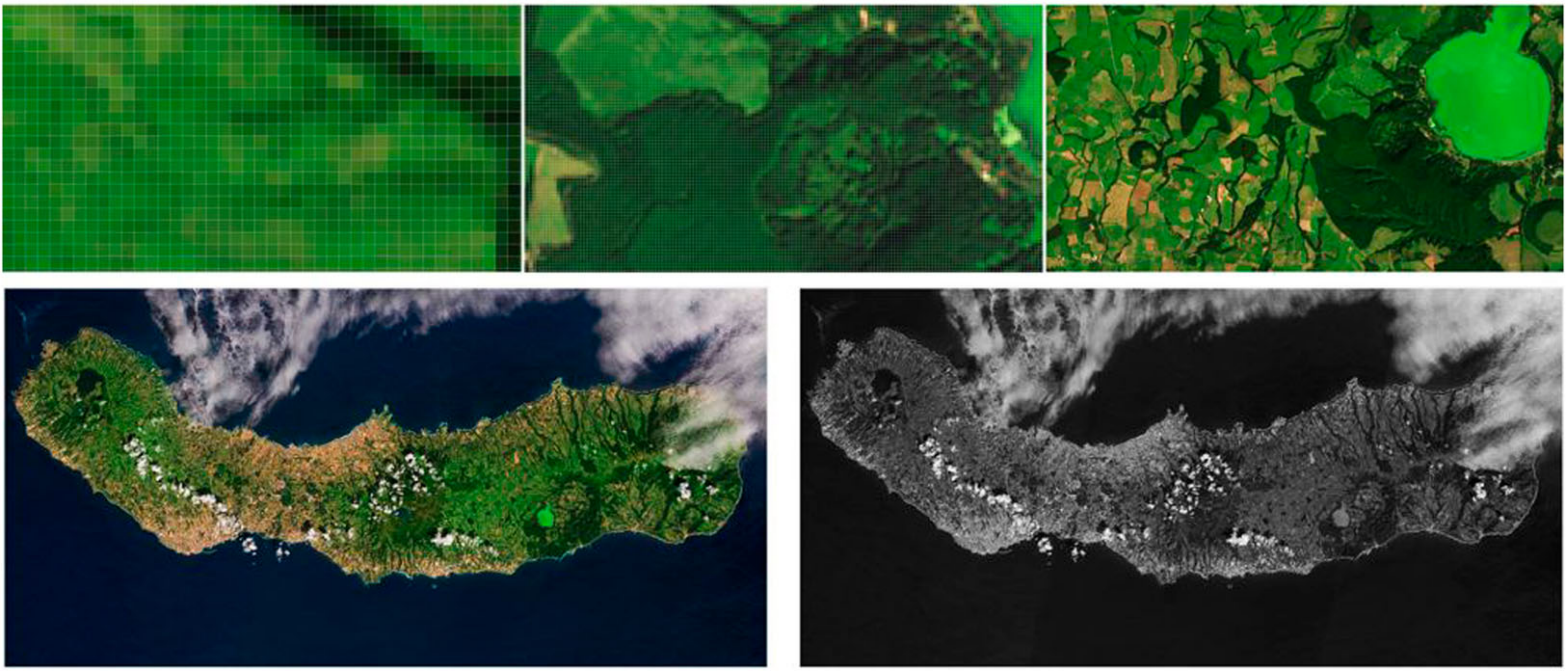
Example image showing that spatial information dominates colour in the interpretation process. The top row shows image subsets at different zoom levels. Although the colour information is always the same, interpretation of the first subset is highly ambiguous. While zooming out, the image content becomes clearer as the zoom level allows identifying the spatially arranged objects. The full view reveals an island, although the north-eastern shore is covered by clouds. The B&W image version holds almost the same information, in particular on this high semantic level. Contains modified Copernicus Sentinel data (2016), processed by ESA, CC BY-SA 3.0 IGO (https://www.esa.int/spaceinimages/Images/2018/09/Sao_Miguel_Azores).

Today’s image analysis techniques may revisit and consider characteristics of (human) vision as pre-requisite. Vision as a cognitive process deals with the semantic information gap between ever-varying sensory data and stable categories in a mental world (Marr 1982). Defined as the process of scene-from-image reconstruction and understanding, vision is an ill-posed problem in the Hadamard sense where a solution does not exist, is not unique, or the solution's behaviour changes continuously with the initial conditions. Framing the feature extraction process requires a world model or ontology, which consists of entities, relationships and events, and is available as prior mental knowledge about the physical world when looking at an image. The spatial component is of particular importance in the image recognition process. Human vision is so powerful because it considers the spatial arrangement (the context) of objects depicted in an image, making use of a mental knowledge of the physical world in the scene-from-image reconstruction and analysis process (see Figure 4).

In the ongoing debate about big Earth data analytics, including currently employed state-of-the-art systems, pixel-based or local window-based 1D image analysis are dominant. Spatial analyses are often limited to statistical analysis and aggregation of a given area of interest, neglecting the true spatial arrangement or 2D context. Spatial image analysis, as promoted by Lang et al. (2018) in reconciliation of the GEOBIA paradigm (Blaschke et al. 2014), is making explicit use of spatial concepts in a convergence of evidence approach comprising contextual, geometrical, topological, and even hierarchical (i.e. scale-related) features. Often, an approach of ‘time first, space later’ to produce information from a temporal stack of images turns into ‘time first, space never’, when remaining in 1D (per-pixel) analysis.

Developments of sensing observations and producing information from it need to be accompanied by suitable storage, processing and retrieval systems. While in the last years data cube has emerged as alternative to 2D raster file-based solutions, some features and structures are still missing. For example, CEOS has set a goal to establish at least 20 operational data cube implementations around the globe by 2022, called ‘Road to 20’ (ODCI 2018), which leads to questions about interoperability and standardisations. There is work in this direction already in progress, e.g. as OGC Coverage or OGC WCPS (Baumann 2017a; Wagemann et al. 2018). Further, current implementations of data cubes do not allow topological and non-topological multi-dimensional relationships or even extraction of complex spatial arrangement of image objects. These types of analyses need to be conducted outside the data cube, thus producing additional overhead in copying data. On the structural side, the uptake of ‘thinking in data cubes’ for EO data instead of images requires sufficient training and education material, provided by an interdisciplinary team consisting of data cube vendors, EO specialists, data analysts and domain experts. To overcome technological-oriented visions, views and developments, the efforts of providing training and tutorial material, talks, and support, e.g. by the Open Data Cube Initiative’s (ODCI) as well as the OGC, such as in the context of Big Data Domain Working Group or organising geospatial tracks in conferences and other initiatives, should play an essential role.

Big Earth data as such is related to Al Gore’s vision of a Digital Earth and could also be seen as a (re-)implementation of it (Boulton 2018). The Digital Earth exists in parallel to the physical Earth with some translating elements between them. For example, an EO sensor translates observations of the physical Earth to the Digital Earth; visualisation techniques communicate the data or derived information back. While sensors on Landsat, Sentinel or other satellites are very mature and undergo constant evolutions, visualisation and communication techniques need more attention, even if already highly acknowledged, for example in the 2017 roadmap of the ODCI (2017), or through the development of the NASA World Wind software. This includes integration of EO-data-derived information into common GIS systems, into smartphones, or even augmented reality or virtual reality devices, allowing them to diffuse into a great variety of domains. The Digital Earth approach also allows solving questions, which gain significance in the context of big Earth data. This includes linking data sets or combining multiple sensor types (Dhu et al. 2017), development of abstract data types, logical views and data models (Camara et al. 2014; Ferreira, Camara, and Monteiro 2014) as well as Web-based processing (Sudmanns et al. 2018; Wagemann et al. 2018).

The big Earth data slogan to ‘bring the user to the data, not the data to the user’ is widely used and mostly understood in a technical sense. Indeed, the degree of interactivity of EO portals increased over the year and allow users to alter or adapt workflows. However, recent developments, such as opening archives and spreading EO data into new domains and application areas, require a meta-perspective on this slogan, which is to introduce and guide (new) users to EO data. To put big Earth data into value in an operational basis, this perspective may accompany the technical developments.

## 5. Conclusion

The quantity of data has been an issue in remote sensing since the community first tried to operationalise information production from satellite images. Coping with big data issues is therefore not new (Barrett and Curtis 1976, 318). In the last few years, terms like big Earth data have emerged and specialised technologies and methods were developed and applied.

In this overview, different and popular systems and portals allowing access or processing of EO data have been summarised. The overview shows an increased degree of interactivity of the portals in the last years. As expected and already mentioned by several authors (Baumann et al. 2016; Esch et al. 2018; Giuliani et al. 2017; Lewis et al. 2017), the overall direction follows the principle of allowing users to directly process EO data Web-based without prior download. Still, the overview presented in this paper also revealed considerable gaps, which we think are important to be closed. Some of them are of general nature in the big data context, including privacy concerns or development of parallelisable algorithms. Others are more specific to EO data and can be split into the sub-domains: (1) technology; (2) data processing; (3) data access; and (4) applications and users. In the technology domain, spatial analysis of EO data may include spatial, temporal and semantic relations of semantic image objects. These use-cases evolve with the advent of increasingly sophisticated object-extraction techniques (e.g. Convolutional Neural Networks). Still, it requires *a priori* or domain knowledge for setting the frame in which such an image understanding system can work. Further, the work on cross-data-cube processing, e.g. across neighbouring data cubes, and data-cube-agnostic processing chains need to be continued. In the data processing domain, the potential of ARD may be unfolded by either finding a common understanding or communicating clearly the ARD’s differences, individual characteristics, and suitability for tasks. This requires work on defining quality indicators not only for end products, but also for workflows and new ways of reporting them. As we have shown, there are lots of portals with the aim to facilitate data access, but in the end, users struggle to produce information and put it in the context of their question. Techniques, which allow content-based image retrieval and estimation of suitability of data for a specific task may be the first relevant steps. It is also necessary to increase the transparency and be clear about the data availability, completeness, and spatio-temporal distribution of images in the archives of the different portals. To foster EO data usage in various application domains, which is one of the main targets of the European Copernicus programme (Copernicus Programme 2017, 21–23), it needs to be acknowledged that end-users are increasingly coming from a non-EO or even non-spatial domains, which affects user-centric technology developments. Techniques, which support visualisation of temporal changes and uncertainty, as well as skill transferability and competency development can be named here. The use of EO data in the political decision-making process is desirable, but also adds more complexity and importance to the trustworthiness of workflows (Craglia and Nativi 2018). For the development of business models, the reliability of data provision and services, as well as continuity in software development and support, is crucial.

Scientific EO satellites capture data regularly from the Earth’s surface without knowing of what it might contain or what it might be used for. This treasure of data may once disclose finding, which are currently beyond our imagination. ‘Finding’ is the activity of identifying the unknown and making sense of it without putting it into known categories. Big Earth data with its emerging technologies and approaches may be an evolution of EO, but it also may allow us to revolutionise the way we use and apply EO in the various domains.
